# Cost-effectiveness of liraglutide versus lixisenatide as add-on therapies to basal insulin in type 2 diabetes

**DOI:** 10.1371/journal.pone.0191953

**Published:** 2018-02-06

**Authors:** Åsa Ericsson, Divina Glah, Maria Lorenzi, Jeroen P. Jansen, Adam Fridhammar

**Affiliations:** 1 Diabetes Marketing, Novo Nordisk Scandinavia AB, Malmö, Sweden; 2 Formerly: Health Economics & Outcomes Research, Novo Nordisk Ltd, Gatwick, United Kingdom; 3 Evidence Synthesis and Decision Modelling, Precision Health Economics, San Francisco, CA, United States of America; 4 Research Manager, The Swedish Institute for Health Economics, Lund, Sweden; McMaster University, CANADA

## Abstract

**Background:**

We assessed the cost-effectiveness of the glucagon-like peptide 1 receptor agonists liraglutide 1.8 mg and lixisenatide 20 μg (both added to basal insulin) in patients with type 2 diabetes (T2D) in Sweden.

**Methods:**

The Swedish Institute for Health Economics cohort model for T2D was used to compare liraglutide and lixisenatide (both added to basal insulin), with a societal perspective and with comparative treatment effects derived by indirect treatment comparison (ITC). Drug prices were 2016 values, and all other costs 2015 values. The cost-effectiveness of IDegLira (fixed-ratio combination of insulin degludec and liraglutide) versus lixisenatide plus basal insulin was also assessed, under different sets of assumptions.

**Results:**

From the ITC, decreases in HbA1c were –1.32% and –0.43% with liraglutide and lixisenatide, respectively; decreases in BMI were –1.29 and –0.65 kg/m^2^, respectively. An estimated 2348 cases of retinopathy, 265 of neuropathy and 991 of nephropathy would be avoided with liraglutide compared with lixisenatide in a cohort of 10,000 patients aged over 40 years. In the base-case analysis, total direct costs were higher with liraglutide than lixisenatide, but costs associated with complications were lower. The cost/quality-adjusted life-year (QALY) for liraglutide added to basal insulin was SEK30,802. Base-case findings were robust in sensitivity analyses, except when glycated haemoglobin (HbA1c) differences for liraglutide added to basal insulin were abolished, suggesting these benefits were driving the cost/QALY. With liraglutide 1.2 mg instead of liraglutide 1.8 mg (adjusted for efficacy and cost), liraglutide added to basal insulin was dominant over lixisenatide 20μg.IDegLira was dominant versus lixisenatide plus basal insulin when a defined daily dose was used in the model.

**Conclusions:**

The costs/QALY for liraglutide, 1.8 or 1.2 mg, added to basal insulin, and for IDegLira (all compared with lixisenatide 20 μg added to basal insulin) were below the threshold considered low by Swedish authorities. In some scenarios, liraglutide and IDegLira were cost-saving.

## Introduction

Poorly controlled diabetes represents a significant health burden for individuals as, over time, elevations in blood glucose concentrations are associated with serious microvascular and macrovascular complications [1, 2]. It is not surprising, therefore, that diabetes also places a high cost burden on societies across the world [3]. Analyses in Sweden undertaken between 1998 and 2005 confirm that total healthcare costs for diabetes are driven to a large extent by diabetes complications [4–6]. Improving the control of diabetes is therefore imperative from both health and cost perspectives. Both the Swedish National Diabetes Register (NDR) and the Swedish National Board of Health and Welfare consider patients with glycated haemoglobin (HbA1c) levels >8.8% (73 mmol/mol) to be a priority group for intensified intervention [7, 8]. This approach is supported by several studies showing that intensive glycaemic control reduces the risk of diabetes-related complications in type 2 diabetes (T2D) [9–12]. Based on a large prospective diabetes study, it has been shown that a reduction in HbA1c levels by one percentage point lowers the risk of diabetes-related death by 21%, overall mortality by 14%, myocardial infarction by 14%, amputation or death from peripheral vascular disease by 43% and microvascular complications by 37% [10]. Research also suggests there will be cost benefits from improved glycaemic control. A retrospective database analysis involving more than 6700 patients in the USA showed that diabetes-related costs for patients who achieved HbA1c levels of ≤7% (55 mmol/mol) were 24% lower than for those with higher HbA1c levels [13].

For patients with T2D uncontrolled on oral antidiabetic drugs (OADs) or glucagon-like peptide 1 receptor agonists (GLP-1RAs), the American Diabetes Association (ADA) and the European Association for the Study of Diabetes (EASD) guidelines recommend the use of basal insulin as an alternative option [14]. Insulin therapy is an established and effective treatment for lowering HbA1c levels in T2D, and often becomes a necessity as endogenous insulin secretion declines. However, insulin treatment is not without its disadvantages, which are principally considered to be weight gain and the risk of hypoglycaemia [14]. Insulin-based treatment regimens may also be complex, and this, together with a fear of adverse effects, such as hypoglycaemia, may adversely impact adherence to treatment [15, 16]. In turn, this can compromise glycaemic control [17]. The number of injections and the need to inject at specific times are commonly reported as difficulties associated with insulin treatment [18], and a Swedish survey conducted in 2008 supports this. In the survey, patients indicated they would be willing to pay SEK159 (EUR13.78 at the time of the study) per month if their diabetes treatment involved one fewer injection per day and SEK140 per month to avoid taking medication with meals [19]. When therapeutic targets can no longer be achieved with basal insulin alone, the addition of a GLP-1RA may be an attractive alternative to the addition of bolus insulin to the regimen [14]. GLP-1RAs are associated with reductions in weight and postprandial glucose excursions, as well as a low risk of hypoglycaemia [14]. A meta-analysis of studies in which patients with T2D received a GLP-1RA with basal insulin has shown the combination provides good glycaemic control without increasing the risk of hypoglycaemia or weight gain [20].

Liraglutide is a GLP-1RA of particular interest because, unlike lixisenatide, it can be injected at any time of the day [21, 22]. Furthermore, the recently completed LEADER study showed that liraglutide significantly reduces the risk of major adverse cardiovascular events in patients with T2D and cardiovascular disease or with cardiovascular risk factors, making it the first GLP-1RA to show this effect [23] (no cardiovascular benefit was shown for lixisenatide in the ELIXA cardiovascular outcomes trial [24]). Additionally, in a head-to-head trial in patients failing to maintain glycaemic control with metformin monotherapy, liraglutide provided similar weight reductions to lixisenatide but with superior glycaemic control [25].

IDegLira was the first fixed-ratio combination of basal insulin and GLP-1RA. Insulin degludec, the basal insulin component, has been shown to provide a consistently low variability in blood glucose levels over 24 hours [26, 27] and IDegLira has been extensively studied in the DUAL clinical trial programme [28]. In a 26-week treat-to-target study in patients not achieving glycaemic control with insulin glargine and metformin, HbA1c reductions with IDegLira were superior to those with uptitrated insulin glargine [29]. IDegLira was also associated with fewer confirmed hypoglycaemic episodes and with weight loss rather than weight gain. In a separate study, IDegLira provided glycaemic control superior to that of insulin degludec at equivalent insulin doses, without a higher risk of hypoglycaemia and with the benefit of weight loss [30]. IDegLira, like liraglutide, can also be administered once daily at any time of the day [31].

It is important to establish the implications of adding a GLP-1RA to basal insulin. Here, we evaluate the cost-effectiveness of the once-daily GLP-1RA liraglutide added to basal insulin, compared with once-daily lixisenatide added to basal insulin, for Swedish patients with T2D, using a validated cost-effectiveness model. As no head-to-head studies comparing the two combinations were available, an indirect treatment comparison (ITC) was performed to obtain the relative treatment effects, for use in the model. We also report additional analyses evaluating the cost-effectiveness of the fixed-ratio combination of liraglutide and insulin degludec (IDegLira) under four different sets of assumptions.

## Methods

### Decision problem

This analysis aimed to assess the cost-effectiveness of the GLP-1RAs liraglutide and lixisenatide, both added to basal insulin, or the fixed-ratio combination IDegLira, in a population comprising adults with T2D and inadequate glycaemic control (HbA1c>7% [53 mmol/mol]) after at least 3 months of basal insulin treatment, from a societal perspective in Sweden. The analysis was performed with a 3% discount rate on costs and effects and a 40-year time horizon (to capture all costs and effects for the remainder of the patient’s life).

### Model structure and assumptions

Analyses were performed using the validated IHE Cohort Model of Type 2 Diabetes developed by the Swedish Institute for Health Economics [32]. This model has been used previously to compare the cost-effectiveness of liraglutide versus sulphonylurea or sitagliptin in Swedish patients, and to compare GLP-1RAs, dipeptidyl peptidase-4 (DPP-4) inhibitors, and neutral protamine Hagedorn (NPH) insulin [33, 34]. The National Board of Health and Welfare also used the model in the development of the 2015 National Guidelines for Diabetes Care [35].

In brief, the model uses two parallel Markov chains. The first process comprises 120 microvascular health states combining different stages of retinopathy, neuropathy and nephropathy (based on previously published work [36–38]). The second process comprises 100 macrovascular health states combining different stages of ischaemic heart disease, myocardial infarction, stroke and heart failure (based on the United Kingdom Prospective Diabetes Study [UKPDS] Outcomes Model 1 [39]). The model permits a choice of risk equations and uses a wide range of variables, including cohort baseline characteristics, treatment algorithms, unit costs and utility weights. Baseline characteristics encompass demographics, biomarkers (such as body mass index [BMI], HbA1c level, blood pressure and serum lipid level) and any complications present at baseline. The model can yield analyses specific to men or women and smokers or non-smokers, and can be conducted with a society or healthcare perspective. It has a cycle length of 1 year and a maximum time horizon of 40 years. Outcomes include cumulative incidences of complications and adverse events, the number of years of survival, quality-adjusted life-years (QALYs), costs, incremental cost-effectiveness ratios (ICERs, expressed as cost/QALY) and net monetary benefits. Both health gains and costs may be discounted. Further details on the structure of the model are shown in the **Supplementary Methods A in S1 File** and in the validation study [32].

A number of specific assumptions were made for the cost-effectiveness analyses reported here. Swedish NDR equations [40] were employed to model the development of macrovascular complications, and risk equations from the UKPDS Outcomes Model 2 were used for mortality [41]. In the treatment algorithms, HbA1c levels declined from starting values during the first year; thereafter, HbA1c levels increased slowly by 0.15% per annum (based on long-term data from the UKPDS [42, 43]). When HbA1c levels reached the predetermined threshold of 8.8% (the threshold defined by Swedish authorities as a priority group for intensified intervention, as described above), treatment was switched to a basal–bolus regimen. For cardiovascular risk factors, it was assumed that a daily 20 mg dose of an angiotensin-converting enzyme inhibitor would be administered for systolic blood pressure values ≥140 mmHg and would reduce values by 5%. Similarly, it was assumed that daily 40 mg doses of adjunct statins would be administered and would reduce levels of low-density lipoprotein cholesterol ≥2.5 mmol/L by 20%, and that fibrate treatment would reduce triglyceride levels ≥1.7 mmol/L by 20%.

### Treatment effects

As outlined in the *Decision problem*, studies met the inclusion criteria if they were conducted in adult patients with T2D and HbA1c levels >7% (53 mmol/mol) despite ≥3 months of treatment with basal insulin, and if they compared liraglutide + basal insulin or lixisenatide + basal insulin with basal insulin alone. The endpoints of interest were changes in HbA1c, fasting plasma glucose and weight; the proportions of patients achieving HbA1c targets (<7% or ≤7%); and the rates of hypoglycaemic events (severe, mild/non-severe and overall, expressed as events per patient per year). These studies were identified from a literature search conducted in November 2014 (**Supplementary Methods A in S1 File**). Two randomised controlled trials (RCTs) were retrieved: LIRA-ADD2BASAL [44] (full results were published in 2015) and the GetGoal-L study [45]. These studies had the same basic design: they were both randomised, double-blind, placebo-controlled studies conducted in patients with T2D and inadequate glycaemic control, defined in both cases as HbA1c levels of 7–10% (53–86 mmol/mol), while receiving basal insulin with or without metformin [44, 45]. The studies had similar durations (26 weeks for LIRA-ADD2BASAL and 24 weeks for GetGoal-L) and baseline characteristics (duration of diabetes, distribution of race/ethnicity, weight and baseline HbA1c levels). In the LIRA-ADD2BASAL study, patients received liraglutide 1.8 mg (n = 226) or placebo (n = 225) added to insulin glargine (67% of patients) or insulin detemir (33%). In the GetGoal-L study, patients received lixisenatide 20 μg (n = 328) or placebo (n = 167) administered once daily 1 hour before breakfast, added to insulin glargine (50% of patients), NPH (40%) or insulin detemir (9%). A further eight patients received a premix insulin, although this was a protocol violation.

Where possible, cohort baseline characteristics for the cost-effectiveness analyses were derived from the LIRA-ADD2BASAL study [44]; data not reported in the primary publication of this study were derived instead from the DUAL II study [30] (**Table 1**). The DUAL II study was a 26-week, randomised, double-blind comparison of IDegLira and IDeg (both in combination with metformin) in patients with T2D and inadequate glycaemic control (HbA1c 7.5–10.0% [58–86 mmol/mol]) while receiving basal insulin and OADs [30]. Inclusion criteria for DUAL II were similar to those for LIRA-ADD2BASAL, and baseline demographic characteristics reported in both studies were similar enough to justify this approach (Table 1). Additional baseline variables that were not derived from clinical studies are described in the **Supplementary Methods A in S1 File**.

**Table 1:** Baseline values derived from the LIRA-ADD2BASAL and DUAL II* studies [30, 44].

	LIRA-ADD2BASAL	DUAL II
Age at start, years	59.3 (9.2)	57 (9) and 58 (11)^a^
Diabetes duration, years	12.1 (7.1)	10 (6) and 11 (7)^a^
Body mass index, kg/m^2^	32.3 (5.6)	33.6 (6) and 33.8 (6)^a^
Glycated haemoglobin		
%	8.2 (0.8)	8.7 (0.7) and 8.8 (0.7)^a^
mmol/mol	66.3 (8.8)	72 (8) and 73 (8)^a^
Systolic blood pressure (mmHg)		132.4 (15.1)^b^
Cholesterol (mmol/L)^†^		
Total		4.67 (1.21)^b^
Low-density lipoprotein		2.58 (0.96)^b^
High-density lipoprotein		1.16 (0.31)^b^
Triglycerides (mmol/L)^†^		2.18 (2.10)^b^

Data are means (SD).

^a^Values for the IDegLira and IDeg groups in DUAL II are shown for the purposes of comparison only

^b^DUAL II data are not previously published.

^†^Data have been converted from mg/dL to mmol/L by dividing by 39 (cholesterol) or 89 (triglycerides). SD, standard deviation.

Treatment effects for liraglutide added to basal insulin for the base-case analysis were derived from the LIRA-ADD2BASAL study. An ITC was performed to obtain the treatment effects for lixisenatide added to basal insulin relative to liraglutide added to basal insulin because there were no head-to-head or existing ITC studies for the these two combinations **(Supplementary Methods A in S1 File)**. The ITC was facilitated by similarities in the placebo arms of the two studies, which were combined into a single node, and similarities in the distributions of effect modifiers. Estimated differences in reductions in HbA1c levels and body weights obtained by the ITC for lixisenatide versus liraglutide (both added to basal insulin) were –0.89% (95% confidence interval [CI]: –1.23;–0.54) and –1.81 kg (95% CI: _2.83;–0.78), respectively (lower reductions in HbA1c and body weight with lixisenatide). From this point onwards within this manuscript, weights are to be considered in terms of BMI as an input utility, having taken into account the mean patient height. All other clinical inputs (systolic blood pressure, lipids and hypoglycaemic events) were considered equal between treatments **(Table A in S1 File)**.

### Additional scenario analyses for IDegLira versus lixisenatide added to basal insulin

The cost-effectiveness of IDegLira was compared with that of lixisenatide added to basal insulin under four different ‘scenarios’ or sets of assumptions. The efficacy of IDegLira was derived from a published indirect pooled comparison of IDegLira versus three other strategies for treating patients with type 2 diabetes inadequately controlled on basal insulin, including the addition of liraglutide to basal insulin [46]. Further details are provided in the **Supplementary Methods A in S1 File (Table B in S1 File)**.

### Resource use and costs

Resource use and cost inputs are described in brief below, with additional detail provided in the **Supplementary Methods A in S1 File**.

Daily doses were derived from the LIRA-ADD2BASAL and GetGoal-L studies [44, 45] (**Table C in S1 File**). All patients were assumed to be receiving metformin 1500 mg daily, as an adjuvant to study medication and insulin glargine as the basal insulin. The costs of drugs and consumables were based on pharmacy retail prices, excluding VAT, listed in the Swedish Dental and Pharmaceutical Benefits Agency price database in July 2016 (**Table C in S1 File**). Average treatment costs used in the analyses are shown in **Table D in S1 File**.

Costs associated with hypoglycaemia and diabetes-related complications, as well as indirect costs, are reported in 2015 values (adjusted upwards from 2013 and 2014 values by 0.7% in line with the Swedish healthcare consumer price index, as necessary; data for adjustment to 2016 values were not available). Costs associated with hypoglycaemia were calculated from data derived from Swedish patients with T2D [47, 48] as described in the **Supplementary Methods A** and **Table E and Table F in S1 File**. The costs of complications were identified in a literature review completed as part of a cost-effectiveness analysis of liraglutide compared with sitagliptin and sulphonylurea [33] (**Table G in S1 File**). Data on days absent from work due to diabetes-related complications were obtained from a Danish registry analysis that comprised 34,882 patients with diabetes (of whom 14,746 were working) [49] (**Supplementary Methods A** and **Table H in S1 File**).

### Utilities

The model accounted for the impact of a range of factors: demographics (age, gender, and duration of diabetes), treatment (number of injections, regimen complexity and blood glucose tests), treatment effects on clinical factors (HbA1c, BMI and hypoglycaemia) and long-term micro- and macrovascular complications (see **Supplementary Methods** and **Tables I–L in S1 File**). In accordance with the Swedish National Board of Health and Welfare, cost/QALY values below SEK500,000 in the present study were considered to be ‘moderate’ and therefore cost-effective [50]. Cost/QALY values below SEK100,000 are considered ‘low,’ those between SEK500,00 and SEK1,000,000 are ‘high,’ and those above SEK1,000,000 are ‘very high’.

### Sensitivity analyses

A probabilistic sensitivity analysis (PSA) was conducted to assess the uncertainty of the input parameters using a Monte Carlo simulation with 1000 iterations. This was then used to calculate the mean and 95% confidence intervals of QALYs, costs and costs/QALY. Standard errors (SEs) for treatment effects were derived from the LIRA-ADD2BASAL and GetGoal-L studies [44, 45]. SEs for costs associated with micro- and macrovascular complications were set to 10% of the mean value, while SEs were set to 20% of the mean value for costs associated with hypoglycaemia, and an SE of 0% was adopted for pharmaceutical pricing. Patient benefits from treatment were varied within a range of 50% from base-case values. SEs for QALYs associated with hypoglycaemia were derived from published data [51], as were SEs for QALYs associated with neuropathy and nephropathy [52], whereas QALYs for all other diabetes-related complications were varied within a range of 10% from base-case values (**Table M in S1 File**).

A series of univariate sensitivity analyses were conducted to assess the impact of variations in model assumptions and inputs on the base-case analysis. Alternative time horizons considered were 10, 20 and 30 years and alternative discount rates for QALYs and costs were 0 and 5%. Sensitivity analyses also included an HbA1c threshold for intensification of 8.2% instead of 8.8% and the abolition of two key outcome drivers (differences in HbA1c levels and BMI between treatments) or the use of the upper and lower bounds of the 95% CIs for these effects. Further sensitivity analyses examined the use of alternative treatment costs (using a healthcare perspective and cost data for liraglutide 1.2 mg [rather than 1.8 mg] and the intermediate-acting basal insulin Insuman^®^ basal [rather than insulin glargine]). In the sensitivity analysis using liraglutide 1.2 mg, the price was decreased according to the pharmacy selling price in Sweden); the decrease in HbA1c was changed to 0.98%; and the decrease in weight was changed from BMI –1.27 to BMI –1.07. These values for the efficacy of liraglutide 1.2 mg relative to liraglutide 1.8 mg were derived from an RCT of patients with T2D uncontrolled on OADs in which each dose was compared with glimepiride (all administered as monotherapy) [53]. Alternative costs of diabetes-related complications considered were +20 and –20% and an analysis was conducted with all patient utilities from treatment and short-term HbA1c disutility removed. Non-smoking men are considered as the base-case scenario for the purpose of this analysis. A change in gender or smoking status did not affect the results and are not considered further in this manuscript.

The current analysis used the costs for IDegLira at maximum dose (1.8 mg liraglutide and 50 IU basal insulin) or at defined daily dose (i.e. the assumed average maintenance dose/day for a drug used for its main indication in adults [54]; for IDegLira, the defined daily dose was calculated as 1.44 mg liraglutide plus 40 IU basal insulin).

Two different efficacy assumptions were made for each of the two dosing levels. In the first scenario, the efficacy of IDegLira was conservatively assumed to be equivalent to that of liraglutide 1.8 mg added to 36 IU basal insulin, even though the insulin dose in IDegLira was higher (50 or 40 IU). In the second scenario, IDegLira was assumed to have added efficacy compared with liraglutide added to basal insulin, based on the published pooled analysis [46].

## Results

### Cost-effective analyses of liraglutide versus lixisenatide (each added to basal insulin)

#### Base-case analysis

The number of complications avoided in a cohort of 10,000 patients treated with liraglutide versus lixisenatide (each added to basal insulin) over 40 years is shown in **Table 2**.

**Table 2:** Number of complications avoided in a cohort of 10,000 patients over 40 years.

Event	Number avoided with liraglutide versus lixisenatide
Retinopathy	2348
Neuropathy	265
Nephropathy	991
Myocardial infarction	95
Stroke	156
Congestive heart failure	368

**Table 3** shows health gains, costs, costs/QALY and the net monetary benefit for liraglutide versus lixisenatide (each added to basal insulin) in the base-case analysis. Although the total direct costs of liraglutide added to basal insulin were higher than those for lixisenatide added to basal insulin, direct costs associated with complications, particularly for nephropathy, and indirect costs (i.e. production losses) were lower. The cost/QALY for liraglutide added to basal insulin was SEK30,802/QALY. The cost/QALY was well below the threshold of SEK100,000/QALY seen to represent a ‘low’ cost/QALY assumed in the present study to represent cost-effectiveness in Sweden.

**Table 3:** Base-case analysis of liraglutide 1.8 mg compared with lixisenatide 20 μg (each added to insulin glargine).

	Liraglutide 1.8 mg (added to basal insulin)	Lixisenatide 20 μg (added to basal insulin)	Increment for liraglutide versus lixisenatide (each added to basal insulin)
**Health gain**			
Survival after 40 years	–	–	–
Life-years	13.35	13.17	0.18
QALYs	6.57	5.71	0.86
**Direct costs (SEK)**			
Background costs	0	0	0
Anti-hyperglycaemic treatment			
Treatment	305,067	233,179	71,888
Hypoglycaemia	1192	1980	–788
Other adverse events	0	0	0
Other treatments			
Hypertension	0	0	0
Dyslipidaemia	19,517	19,259	258
Obesity	0	0	0
Microvascular complications			
Retinopathy	8483	14,302	–5819
Neuropathy	67,406	71,425	–4018
Nephropathy	111,636	132,725	–21,088
Macrovascular complications			
Ischaemic heart disease	27,855	28,371	–517
Myocardial infarction	24,606	25,364	–758
Stroke	136,441	144,189	–7748
Congestive heart failure	32,442	35,824	–3382
Total direct costs	734,646	706,618	28,028
**Indirect costs (SEK)**			
Production loss	22,120	23,734	–1614
Net consumption	0	0	0
**Total direct and indirect costs**	**756,766**	**730,352**	**26,414**
**Cost-effectiveness of liraglutide 1.8** **mg versus lixisenatide (each added to insulin glargine)**			
Cost per life-year	SEK149,609/life-year		
Cost per QALY	SEK30,802/QALY		

Base-case with 3% discount rate. All costs are described in SEK. QALY, quality-adjusted life-year; SEK, Swedish kronor.

**Fig 1:**
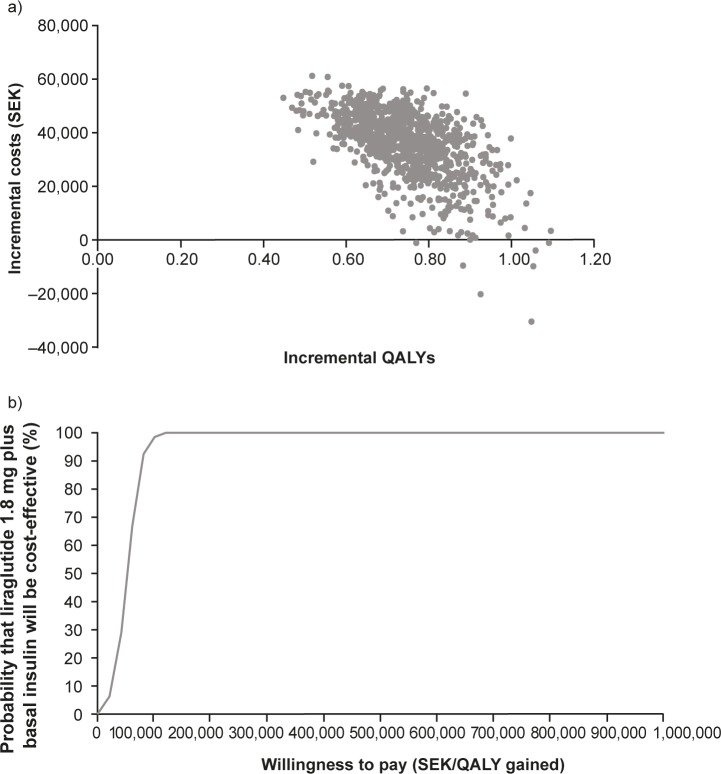
a) Cost-effectiveness plane and b) cost-effectiveness acceptability curve for liraglutide 1.8 mg versus lixisenatide (each added to basal insulin). Probabilistic sensitivity analysis conducted with 1000 simulations. Base-case with 3% discount rate. QALY, quality-adjusted life-year; SEK, Swedish kronor.

**Fig 1** shows the cost-effectiveness plane and the cost-effectiveness acceptability curve from the PSA. Overall, 99% of the estimated costs/QALY were within the north-east quadrant of **Fig 1A**, indicating that liraglutide 1.8 mg added to basal insulin had higher benefits and higher costs than lixisenatide added to basal insulin. At a willingness-to-pay value of SEK500,000/QALY, liraglutide added to basal insulin was cost-effective in 1000 out of 1000 simulations (100%) (**Fig 1B**).

#### Additional sensitivity analyses

Overall, the univariate sensitivity analyses showed that the base-case analysis was robust in the face of variation in key model inputs and assumptions (**Table 4**). The exception was the analysis in which the HbA1c difference between treatments was abolished; in this case, the cost/QALY rose to SEK817,900. This implies that the treatment difference in HbA1c reductions is the driver of the results. When liraglutide 1.2 mg was substituted for liraglutide 1.8 mg in one of the sensitivity analyses, liraglutide 1.2 mg added to basal insulin was dominant over lixisenatide 20 μg added to basal insulin.

**Table 4:** Sensitivity analyses for liraglutide 1.8 mg compared with lixisenatide 20 μg (each added to basal insulin).

Base-case scenario	ΔCosts (SEK)	ΔQALYs	Cost/QALY (SEK)^a^
Liraglutide 1.8 mg versus lixisenatide (each added to insulin glargine)	26,414	0.86	30,802
**Alternative time horizons**			
30 years	25,829	0.85	30,521
20 years	39,231	0.77	50,964
10 years	46,487	0.48	97,075
**Alternative discount rates**			
0%	13,210	1.21	10,901
5%	30,329	0.69	43,769
**Different assumptions for HbA1c**			
Treatment intensification at HbA1c 8.2% instead of 8.8%	13,396	0.93	14,357
HbA1c progression for GLP-1 = 0.08%	44,566	0.88	50,459
**Key outcome drivers abolished or changed**			
Between-treatment difference in HbA1c abolished	76,326	0.09	817,877
HbA1c difference between treatments, 95% CI lower bound	43,368	0.63	68,528
HbA1c difference between treatments, 95% CI upper bound	4,637	1.06	4,637
Between-treatment difference in BMI abolished	26,511	0.81	32,668
BMI difference between treatments, 95% CI lower bound	26,470	0.83	32,017
BMI difference between treatments, 95% CI upper bound	26,360	0.91	29,017
**Alternative treatment costs**			
Healthcare perspective	28,028	0.86	32,591
Liraglutide 1.8 mg replaced with 1.2 mg^b^	–13,996	0.58	Liraglutide dominant
Insulin glargine replaced with an intermediate-acting basal insulin (Insuman^®^ basal)	22,540	0.86	26,285
**Alternative complication costs**			
+20%	17,790	0.86	20,746
−20%	35,080	0.86	40,908
**Alternative assumptions about patient utility**			
No patient utility from treatment (flexibility, injections procedures and SMBG) and short-term HbA1c utility	26,414	0.32	81,601

^a^As the cost/QALY is calculated directly from individual values in the model, the results are not necessarily identical to those seen when dividing mean Δcosts by mean ΔQALY values.

^b^Relative efficacy derived from LEAD 3. Prices for drugs were obtained from the Swedish Dental and Pharmaceutical Benefits Agency price database in July 2016. All other costs are reported in 2015 values. Δ, difference in; BMI, body mass index; HbA1c, glycated haemoglobin; QALY, quality-adjusted life-year; SMBG, self-monitoring of blood glucose.

### Cost-effectiveness analyses of IDegLira versus lixisenatide 20 μg added to basal insulin

Results of the additional analyses comparing IDegLira with lixisenatide added to basal insulin are shown in **Table 5**.

**Table 5:** Cost/QALY for additional scenarios evaluating the cost-effectiveness of IDegLira compared with lixisenatide 20 μg added to basal insulin.

Scenario	Dose	Efficacy	Cost/QALY (SEK)
1	Maximum	Equivalent to liraglutide 1.8 mg added to insulin glargine 36 IU	34,800
2	Maximum	Added efficacy compared with liraglutide added to basal insulin (derived from pooled analysis [54])	23,984
3	Defined daily dose	Equivalent to liraglutide 1.8 mg with insulin glargine 36 IU	IDegLira dominant
4	Defined daily dose	Added efficacy compared with liraglutide added to basal insulin (derived from pooled analysis [54])	IDegLira dominant

Maximum dose: 1.8 mg liraglutide and 50 IU basal insulin. Defined daily dose: 1.44 mg liraglutide plus 40 IU basal insulin.

In the most conservative scenario (using the costs associated with the maximum dose of IDegLira and with IDegLira efficacy equivalent to that of liraglutide 1.8 mg added to basal insulin [scenario 1]), the cost/QALY was very similar to that for liraglutide 1.8 mg added to basal insulin in the base-case analysis (SEK34,800 vs. SEK30,802, respectively). Accordingly, IDegLira was below the threshold of SEK100,000/QALY seen to represent a ‘low’ cost/QALY. In the scenario accounting for the added efficacy of IDegLira over liraglutide added to basal insulin, and retaining the costs associated with the maximum IDegLira dose (scenario 2), the cost/QALY was reduced (**Table 5**). In the remaining two scenarios, both of which used the costs of the defined daily dose of IDegLira, IDegLira was dominant over lixisenatide added to basal insulin.

## Discussion

This is the first analysis of the cost-effectiveness of liraglutide 1.8 mg compared with lixisenatide 20 μg, each added to basal insulin, in patients with T2D uncontrolled on basal insulin. In the absence of head-to-head trials with these two therapies, an ITC was used to determine relative treatment effects. The ITC showed that liraglutide 1.8 mg added to basal insulin was more efficacious than lixisenatide 20 μg added to basal insulin in terms of improved glycaemic control and weight loss. With the relative treatment effects (specifically changes in HbA1c levels and body weight) incorporated into the model, liraglutide 1.8 mg added to basal insulin was associated with a cost/QALY in a Swedish setting of SEK30,802 relative to lixisenatide 20 μg added to basal insulin. This was well below the threshold of SEK100,000/QALY considered to be cost-effective by Swedish authorities. In the univariate sensitivity analyses, costs/QALY were consistent with those from the base-case, with two exceptions. When the efficacy benefits in terms of HbA1c levels were abolished, the cost/QALY increased. When liraglutide 1.2 mg was substituted for liraglutide 1.8 mg (each added to basal insulin), the 1.2 mg combination was dominant (less costly and more effective) over lixisenatide 20 μg added to basal insulin. The IDegLira costs/QALY (relative to lixisenatide 20 μg added to basal insulin) were well below the threshold considered to represent cost-effectiveness in all of the tested scenarios. In less conservative scenarios (based on more realistic dosing and efficacy assumptions), IDegLira was dominant over lixisenatide 20 μg added to basal insulin.

The ADA–EASD position statement emphasises the importance of individualising both treatment targets and treatment strategies [14]. In the absence of head-to-head RCT evidence regarding the comparison of interest, indirect comparisons are commonly used to derive the relative treatment effects for comparators of interest. In the case of the once-daily GLP-1RAs liraglutide and lixisenatide, however, information is limited. A recent head-to-head clinical trial involving liraglutide 1.8 mg and lixisenatide 20 μg as add-ons to metformin in patients with T2D failing to maintain glycaemic control with metformin alone showed that liraglutide provided weight reductions similar to lixisenatide but superior glycaemic control [25]. No similar trials have been undertaken for these two agents added to basal insulin. The ITC presented here provides insights into treatment differences that may be of interest to clinicians. This ITC is also supported by the treatment difference in favour of liraglutide observed in the above-mentioned liraglutide versus lixisenatide RCT [25]. Although that trial was conducted in an OAD-failure population, there is no evidence to suggest that the improved efficacy of liraglutide over lixisenatide would not be seen in a basal insulin-failure population.

Notwithstanding the relevance of the data to clinical practice, the ITC was primarily performed to facilitate cost-effectiveness evaluations. The superiority of liraglutide 1.8 mg in terms of glycaemic control that was evident in the ITC was a key driver of differentiation in terms of value. The cost/QALY of liraglutide 1.8 mg relative to lixisenatide 20 μg (each added to basal insulin) was robust in all sensitivity analyses, except when efficacy differences (HbA1c levels) were abolished.

The costs/QALY yielded by the analyses presented here indicate that liraglutide 1.8 mg added to basal insulin is a cost-effective treatment compared with lixisenatide 20 μg added to basal insulin in patients with T2D uncontrolled with basal insulin in Sweden. The base-case analysis was conducted with liraglutide 1.8 mg because this was the dose in the clinical trial used in the ITC. In Sweden, however, the majority of patients are prescribed the 1.2 mg dose [55]. In a sensitivity analysis conducted with this dose, liraglutide 1.2 mg added to basal insulin was the dominant treatment compared with lixisenatide 20 μg. This suggests that, for most patients with T2D uncontrolled on basal insulin in Sweden, the addition of liraglutide 1.2 mg is cost-saving compared with the addition of lixisenatide 20 μg. A possible limitation is that, in the current analysis, the efficacy of the 1.2 mg dose was based on the results of a trial of liraglutide as monotherapy [53], as no studies are available of the 1.8 mg dose plus basal insulin versus the 1.2 mg dose plus basal insulin. However, there is no evidence to suggest that the treatment difference would not be achieved in patients with T2D uncontrolled with a basal insulin. It is, moreover, a conservative choice of trial in the context of these cost-effectiveness analyses, as the difference in efficacy between doses was greater in this RCT than in the other LEAD trials [56, 57].

IDegLira, the fixed-ratio combination of insulin degludec and liraglutide, offers greater flexibility in the timing of administration and is a simpler treatment option than lixisenatide added to insulin glargine, due to fewer daily injections as well as the potential for lower doses of basal insulin. In all of the additional scenario analyses presented here, IDegLira was shown to be cost-effective compared with lixisenatide added to insulin glargine. In fact, the costs/QALY yielded were lower than those reported for liraglutide 1.8 mg added to insulin glargine (also relative to lixisenatide added to insulin glargine) in three out of four analyses. It is important nonetheless to note that all scenarios were, to some extent, conservative and the resultant costs/QALY may thus still have been overestimated. The efficacy of IDegLira was underestimated in two analyses by assuming equivalence to a combination therapy with a lower dose (by 14 IU) of basal insulin. Increased insulin doses may lead to improved reductions in HbA1c levels (along with an increased risk of hypoglycaemia). Moreover, in the scenario considered most favourable for IDegLira (in which IDegLira was dominant over lixisenatide added to basal insulin), IDegLira dosing costs were assumed to be equivalent to those of the defined daily dose, an average maintenance dose/day, as defined by the World Health Organization Collaborating Centre for Drugs Statistics Methodology [54]. This defined daily dose is, in fact, higher than the dose of IDegLira in the pooled analysis from which the corresponding efficacy data for the analysis were derived [46].

There are limitations associated with the analyses presented here. As the inclusion criteria were strict, only one trial was included for each intervention of interest and it was therefore not possible to use random-effects modelling for study-based heterogeneity in treatment effects. In the fixed-effect models used instead, credible intervals were likely to have been narrower than they would have been with random-effects modelling, underestimating the uncertainty concerning relative treatment effects. For severe hypoglycaemia, no events were reported in three out of the four treatment arms included in the ITC, precluding stable estimation of relative treatment effects and limiting conclusions that could be drawn in this regard. As with any ITC, unmeasured or unreported differences in patient characteristics between studies may also have affected relative treatment effects. It is important to note that, for the purposes of the ITC, it was assumed that all patients received the same basal insulin. In fact, 33% patients in the liraglutide study received insulin detemir [44], and three different basal insulins were used in the GetGoal-L study (insulin glargine, NPH and insulin detemir) and eight patients received a premix insulin [45]. Nonetheless, in both studies in the ITC, the majority of patients were receiving insulin glargine (GetGoal-L: 50%; LIRA-ADD2BASAL: 67%) [44, 45] and the studies were very similar in terms of other baseline characteristics and trial design. Despite this, the trials were conducted by different sponsors, and indirect comparisons cannot account for trial conduct heterogeneity; insulin being a titratable product with differing titration dose guidelines and maximum doses is a probable source of this heterogeneity.

Any shortcomings in the methodological quality of the trials could affect the reliability of the input values used in the ITC, and thus of the results. As both trials [44, 45] were phase 3 trials conducted by the manufacturers for registration purposes, they were rigorously conducted, meeting high quality standards with respect to trial criteria such as method of allocation, blinding, adequate and properly described statistical methods, measurement of outcomes, and properly described and acceptable dropout rates. The quality of the trials used for the ITC can therefore not be considered a limitation.

However, the use of short-term clinical data to simulate the course of T2D over a 40-year time horizon could be viewed as another limitation of the cost-effectiveness analysis. In the absence of long-term clinical data, this is unavoidable, and it is widely considered standard practice in measuring cost-effectiveness to use a simulation model based on clinical assumptions and long-term risk equations. In this analysis we have also attempted to minimise uncertainty by performing sensitivity analyses and by using conservative assumptions.

The data presented here suggest that both liraglutide 1.8 mg and 1.2 mg added to basal insulin, as well as IDegLira, are clinically effective and cost-effective treatment options, compared with lixisenatide 20 μg added to basal insulin, for patients with T2D uncontrolled on basal insulin in Sweden. In fact, in some scenarios, liraglutide and IDegLira were cost-saving.

## Supporting information

S1 File Supplementary methods and tables. (PDF)
