# Supplementary material for: Cost-effectiveness of liraglutide versus lixisenatide as add-on therapies to basal insulin in type 2 diabetes
Source: PLoS One. 2018 Feb 6;13(2):e0191953. doi: 10.1371/journal.pone.0191953 (PMC5800677; doi:10.1371/journal.pone.0191953)
Supplement: S1 File — (PDF) [file pone.0191953.s001.pdf]

S1 File. Supplementary methods and tables.

Cost-effectiveness of liraglutide versus lixisenatide as add-on therapies to basal insulin in type 2 diabetes

Åsa Ericsson, Divina Glah, Maria Lorenzi, Jeroen P. Jansen and Adam Fridhammar

Supplementary Methods A

Model structure

- In the Institute for Health Economics cohort model of type 2 diabetes (T2D), the user defines one or more treatment algorithms that are determined by glycated haemoglobin (HbA1c) levels. HbA1c levels increase over time at a rate defined by the user and, when they reach a pre-determined threshold, the treatment changes according to the algorithms selected. The dose can then be increased or other medications added.
- The simulation begins with identical cohorts, each commencing one of the treatment algorithms of interest. The development of biomarkers is influenced by the treatment received and is simulated annually for a pre-determined number of years, up to a maximum of 40 years.
- The model takes into account side effects, such as the rate of hypoglycaemia (events per patient per year). This rate is specific to the treatment used in the algorithm but is adjusted automatically as HbA1c levels change.
- The model simulates the development of complications and mortality through annual transitional probabilities. These are influenced by other factors in the model, such as biomarkers and patient demographics [1-5].
- The model can be used with risk equations based on data from either the Swedish National Diabetes Register (NDR) or the United Kingdom Prospective Diabetes Study (UKPDS). Costs and utility weights are applied to the cohort at each annual cycle.
- The model was designed in Microsoft Excel 2013 using Visual Basic for Applications.

Study data used in the analyses

<u>Literature search</u>

A systematic literature search using very broad search terms relating to diabetes and its treatment, undertaken initially in 2012 and last updated in November 2014, was used to identify studies for the present analyses. The databases searched comprised Medline, EMBASE and the Cochrane Central Register of Controlled Trials. A total of 24,579 publications were retrieved. After abstract and full-text screening, 756 relevant T2D publications were identified. Two randomised controlled trials remained after applying the inclusion criteria for the cost-effectiveness analyses; these were included in the cost-effectiveness analyses as described in the Methods section of the main article.

<u>Baseline values</u>

The incidences of cardiovascular disease were generated from the Swedish NDR cohort from which risk equations for macrovascular risk complications were developed (Kiadaliri A, Lund University, Sweden; personal communication). The incidence of atrial fibrillation was not used in the model as it does not impact analyses developed with Swedish NDR equations. Heart rates, white blood cell counts and estimated glomerular filtration rates were derived from the UKPDS cohort from which mortality equations were developed [6].

Indirect treatment comparison (ITC) of liraglutide versus lixisenatide (both added to basal insulin)

- An ITC was performed to estimate the relative treatment effect of liraglutide + basal insulin relative to lixisenatide + basal insulin, based on refs [7] and [8]. Changes from baseline in HbA1c, fasting plasma glucose and weight (subsequently to be considered in terms of BMI as an input utility, having taken into account the mean patient height) were assessed, in addition to the proportions of patients meeting HbA1c target (<7% or ≤7%) and rates per person-year of hypoglycaemic events (overall, mild and severe).
- A regression model with a normal likelihood distribution and identity link was used for continuous outcomes. A binomial likelihood with logit link was used for the proportions of patients meeting HbA1c targets. Hypoglycaemia outcomes were modelled using a Poisson distribution with log link.
- In general, the assumptions of random-effects models are more plausible than those of the fixed-effect model for evidence-synthesis studies. However, the direct comparisons of treatments added to basal insulin were each described by only a single study. In such cases, a heterogeneity parameter cannot be estimated. Accordingly, only results obtained with fixed-effect models are presented for the ITC. In addition, no adjustment was possible to account for other between-trial differences that may affect estimates of relative treatment effects. See **Table A** for clinical input values for treatment effects used in the model.
- The models used to obtain the results are based on publicly available Open Bugs code available from the NICE Decision Support Unit [9].

Table A. Model clinical inputs for treatment effects.

Variable	Liraglutide added to basal insulin [8]	Lixisenatide added to basal insulin	Basal–bolus insulin regimen (rescue treatment in both arms) [10]*
HbA1c (%)†	−1.32	−0.43	−1.33
BMI (kg/m^2)†	−1.3	−0.65	1.38
SBP (mmHg)	−6.86	−6.86	−0.93
TC (mmol/L)	−0.26	−0.26	0.04
LDL (mmol/L)	−0.18	−0.18	0.00
HDL (mmol/L)	0.01	0.01	0.03
Triglycerides (mmol/L)	−0.29	−0.29	0.04
Mild hypoglycaemia (events per person per year)	1.25	1.25	7.95
Severe hypoglycaemia (events per person per year)	0.01	0.01	0.01

Values for liraglutide added to basal insulin were derived from Ahmann *et al.,* 2015 [7].

†Where the differences were statistically signifcant (i.e for HbA1c and BMI), absolute treatment effects for lixisenatide added to basal insulin were calculated by adding the relative treatment effects of lixisenatide versus liraglutide, as obtained with the indirect treatment comparison (previously described herein, based on refs [7] and [8]) to the absolute effects observed in ref. [7]. Where differences were not clinically significant, the change seen with lixisenatide was applied for both treatment arms.

*Values for basal–bolus rescue treatment in both treatment arms are derived from Gough *et al.*, 2014 [10].

BMI, body mass index; HbA1c, glycated haemoglobin; HDL, high-density lipoprotein; LDL, low-density lipoprotein; SBP, systolic blood pressure; TC, total cholesterol.

Indirect pooled comparison of IDegLira versus liraglutide added to basal insulin [11]

- In this published pooled analysis, the efficacy of IDegLira was compared with three other strategies for treating patients with type 2 diabetes inadequately controlled on basal insulin: addition of liraglutide to basal insulin; basal–bolus insulin; or up-titration of insulin glargine.
- The comparisons used individual patient-level data from Novo Nordisk trials with comparable inclusion/exclusion criteria and baseline characteristics. Potential baseline heterogeneity was accounted for using multivariable statistical models [11].
- Data for the addition of liraglutide to basal insulin were derived from Ahmann et al. [7].
- Relative efficacy of IDegLira versus liraglutide added to basal insulin was estimated as shown in S2 Table.

Table B. Estimated efficacy of IDegLira versus liraglutide added to basal insulin, from a pooled analysis [11].

**Variable**	**IDegLira**	**Liraglutide added to basal insulin**
HbA1c (%)	–1.68	–1.33
BMI (kg/m^2)	–1.02	–1.27
Mild hypoglycaemia (events per person per year)	1.22	1.24
Severe hypoglycaemia (events per person per year)	0.004	0

Treatment costs

Resource use and associated prices are shown in **Table C,** based on the LIRA-ADD2BASAL and GetGoal-L studies [7,8]. Pharmacy retail prices, excluding VAT, for drugs and consumables were obtained from the Swedish Dental and Pharmaceutical Benefits Agency database, searched in July 2016 [12]. The prices selected for needles, blood-glucose test strips and lancets were the lowest of those listed in the database. The number of needles used was assumed to equate to the number of injections required in each treatment regimen. The number of test strips used was assumed to be: one for patients receiving glucagon-like peptide 1 receptor agonists added to basal insulin, and four for patients receiving basal–bolus insulin therapy. The unit cost of a blood-glucose test (SEK2.58) was obtained by adding up the prices for a test strip and a lancet. Average treatment costs used in the analyses are shown in **Table D**.

Table C. Resource use and associated prices.

i. Resource use

	**Liraglutide added to basal insulin**	**IDegLira (maximum dose)**	**IDegLira (DDD)**	**Lixisenatide added to basal insulin**	**Basal–bolus insulin regimen***
GLP-1RA	1.8 mg	1.8 mg	1.44 mg	20 ug	–
Basal insulin, IU	35.9	50	40	50	68.2
Bolus insulin, IU	–	–	–	–	57.9
Metformin, mg	1500	1500	1500	1500	1500
Needles	1	1	1	1	4
Test strips	1	1	1	1	4
Lancets	1	1	1	1	4

*Insuman® basal + 3 × Novorapid®. DDD, defined daily dose; GLP-1RA, glucagon-like peptide 1 receptor agonist

ii. Resource prices

	Pack size	Price per pack (SEK)	Price per dose step/dose/piece (SEK)
Liraglutide	54 mg	1500.25	27.78/mg
IDegLira	900 IU IDeg<br>32.4 mg liraglutide	1215.51	1.35/dose step
Lixisenatide	560 µg	621.49	1.11/µg
Insulin glargine	1500 IE	541.49	0.36/IU
Metformin	400 tablets (500 mg)	88.00	0.22/tablet
Insuman® basal	1500 IE	242.31	0.16/IU
NovoRapid®	1500 IE	330.40	0.22/IU
Needles*	100 needles	69.11	0.69/needle
Test strips*	50 strips	116.90	2.34/strip
Lancets*	200 lancets	48.22	0.24/lancet

Prices were obtained from the Swedish Dental and Pharmaceutical Benefits Agency price database (a decision database that provides the prices of specific drugs in reposnse to a query) in July 2016 [12]. *Lowest-priced consumables were supplied by NordicInfu Care AB (needles [I-Fine S 6 mm 31 G] and lancets) and Medtrust Sweden AB (test strips [Wellion LUNA Teststickor]). IDeg, insulin degludec; SEK, Swedish kronor.

Table D. Average treatment costs used in the analysis.

	Liraglutide added to insulin glargine	IDegLira		Lixisenatide added to insulin glargine	Insulin glargine + 3 × insulin aspart*
		Maximum dose	DDD		
Liraglutide	50.01	–	–	–	–
IDegLira	–	67.58	50.38	–	–
Lixisenatide	–	–	–	22.20	–
Insulin glargine	12.96	–	–	18.06	24.63
Insulin aspart*	–	–	–	–	12.75
Metformin	0.66	0.66	0.66	0.66	0.66
ACE inhibitors	0.6	0.6	0.6	0.6	0.6
Statins	0.49	0.49	0.49	0.49	0.49
Fibrate	4.00	4.00	4.00	4.00	4.00
Needles	1.38	0.69	0.69	1.38	2.76
Test strips	2.34	2.34	2.34	2.34	9.35
Lancets	0.24	0.24	0.24	0.24	0.96
Total cost per day	72.98	76.6	59.4	49.97	56.2
**Total cost per year**	**26,638**	**27,959**	**21,681**	**18,239**	**20,513**

Costs obtained from the Swedish Dental and Pharmaceutical Benefits Agency price database (July 2016) [12] and expressed in SEK. The DDD is the assumed average maintenance dose/day for a drug used for its main indication in adults [13]. For IDegLira, the DDD was calculated as 1.44 mg liraglutide plus 40 IU basal insulin.*NovoRapid®. ACE, angiotensin-converting enzyme; DDD, defined daily dose; IDeg, insulin degludec; SEK, Swedish kronor.

Hypoglycaemia

In a report from 2006 using a societal perspective, the costs per severe hypoglycaemic episode for Swedish patients with T2D were estimated to be: EUR63 (cared for by family member only), EUR380 (clinic visit) and EUR3917 (hospitalisation) [14]. Episodes cared for by a family member accounted for 71% of all hypoglycaemia, episodes requiring a clinic visit for 28%, and episodes requiring hospitalisation for 1%. The weighted mean cost per severe (requiring hospitalisation) hypoglycaemic episode used in the analyses reported here were calculated from the study data by converting costs to SEK (assuming EUR1 equates to SEK9.21 in August 2006 values) and adjusting for inflation (using the Swedish healthcare consumer price index). The resultant total cost was SEK1984 (based on 2015 values), comprising healthcare costs of SEK1462 and absenteeism costs of SEK522.

As research has shown that patients with mild hypoglycaemia sometimes seek medical care and there is often some loss of productivity [15], cost data for minor hypoglycaemia were instead derived from data from the Swedish T2D cohort receiving basal insulin therapy in a multinational healthcare resource utilisation study reported in 2013 (**Table E**) [16].

Table E. Healthcare resource utilisation due to mild hypoglycaemia amongst Swedish patients with T2D receiving basal insulin therapy [16].

Patients seeking medical care	Number of additional blood-glucose tests	Absenteeism amongst patients of working age	Absence amongst those reporting
3.6%	1.8	7%†	96.7 minutes*

*This calculation is based on daytime hypoglycaemic events.

†Values (for T2D-BOT in this instance) taken from unpublished study report, not included within the original publication; available on reasonable request from the corresponding author. BOT, basal-supported oral therapy; T2D, type 2 diabetes.

To develop the costs associated with mild hypoglycaemia (**Table F**), it was assumed that half of the healthcare contacts made are with a nurse (SEK580) and half with a physician (SEK1400) [17], giving a mean of SEK990 used as the unit cost for each healthcare contact. The unit cost of blood-glucose tests was obtained by adding up the prices as described earlier. The cost of absenteeism was estimated at SEK184 per hour, with an 2013 average annual income of SEK394,800 for men and SEK340,800 for women [18]; one working year was assumed to consist of 250 working days and one working day of 8 hours. By combining the data on healthcare resource utilisation in **Table E** with the unit costs described above, we were able to calculate the cost per mild hypoglycaemic episode.

The cost of absenteeism was calculated as follows:

Absenteeism amongst patients of working age x *absence in minutes amongst those reporting* x *0.46* x *3.07*

where 0.46 is the proportion of patients of working age in the hypoglycaemia study [16] and 3.07 is the mean income per minute.

Table F. Costs associated with mild hypoglycaemia in Swedish patients with T2D receiving basal insulin therapy.

Healthcare contacts	Additional blood-glucose tests	Total healthcare costs	Absenteeism
35	5	40	10

Costs expressed in SEK. SEK, Swedish kronor; T2D, type 2 diabetes.

Complications

The costs of diabetes-related complications were identified in the literature review completed as part of a cost-effectiveness analysis of liraglutide compared with sitagliptin and sulphonylurea [19], and are shown in **Table G**.

Table G. Annual cost of complications in SEK.

Complication	Description	Costs	Reference(s)
Non-proliferative retinopathy	Visit to ophthalmology clinic	712	[17]
	Screening photography	427	
	Total costs	**1147**	
Proliferative retinopathy	Three visits to ophthalmology clinic (712 per visit)	2136	[17]
	Three visits for laser treatment, including fluorescein angiography (1993 + 6241 per visit)		
	Clinic visit and screening photography in subsequent years	24,702	
	Total costs for first year		
	Total costs for subsequent years	**27,026**	
		**1147**	
Macular oedema	First year: three ranibizumab injections (3 × 12,842)	**38,796**	[17]
	Following year: one ranibizumab injection		
		**12,932**	
Severe visual impairment	First year	**9248**	[20]
	Subsequent years	**3947**	
Symptomatic neuropathy	Gabapentin analgesia (3 × 800 mg/day)	11,746	[12,21]
	Medical treatment of erectile dysfunction (men only)		
	50 mg/week	4936	
	First year (men)	**8399***	
	First year (women)	**5914***	
	Subsequent years (men)	**16,801**	
	Subsequent years (women)	**11,828**	
Peripheral vascular disease	The Eurodiale study: healed wounds	73,581	[22]
	Risk of recurring wounds (assumed 10%)	7358	
	First year	**74,096**	
	Subsequent years	**7410**	
Amputation	The Eurodiale study: major amputation	252,648	[22]
	Risk of recurring wounds (assumed 10%)	7358	
	First year	**254,417**	
	Subsequent years	**7410**	
Microalbuminuria	Treatment with angiotensin receptor blocker		[12]
	(50 mg losartan/8 mg candesartan)	677	
	and calcium antagonist (5 mg)	618	
	First year	**653**	
	Subsequent years	**1304**	

Macroalbuminuria (clinical nephropathy)	Treatment with angiotensin receptor blocker		[12,17]
	(50 mg losartan/ 8mg candesartan)	677	
	and calcium antagonist (5 mg)	618	
	Vitamin D (2 × 500 mg/day)	1312	
	Three physician visits (first year) (3 × 1386)	4158	
	First year	**6812**	
	Subsequent years	**2625**	
Nephropathy (uraemia stage)	KPP database		[23]
	E32 Dialysis	124,584	
	317O Dialysis (primary care; 4085) (3.5 per week over 1 year)	743,470	
	First year (124,584 + 0.5 × 743,470*)	**499,793**	
	Subsequent years	**748,674**	
Ischaemic heart disease (symptomatic)	First year	**94,911**	[24]
	Subsequent years	**3418**	
Myocardial infarction (non-fatal)	First year	**101,468**	[24]
	Subsequent years	**2259**	
Stroke	First year	**181,095**	[25]
	Subsequent years	**162,921**	
Heart failure	First year	**71,449**	[24]
	Subsequent years	**7140**	

*Patients can start treatment at any time during the year; on average, therefore, the first year of treatment will be 6 months in duration. Data from references have been adjusted to 2015 values. SEK, Swedish kronor.

Indirect costs

For the analysis of diabetes-related complications, working age was assumed to be 20–65 years, with an average annual income of SEK394,800 for men and SEK340,800 for women (based on 2013 values; taking into account that an average salary increase of 4.6% since 2013 would marginally affect cost per quality-adjusted life-year (QALY) in favour of liraglutide and IDegLira) [18]. One working year was assumed to consist of 250 working days. Data on days absent from work due to various diabetes complications were obtained from a Danish registry data analysis, which comprised 34,882 patients with diabetes, of whom 14,746 were working [26] (**Table H**). For conditions where it was not possible to determine the number of days absent from work, a conservative assumption was made that no absences occurred. The health economic model does not differentiate between sick days during the 'first' and 'subsequent' years for each condition; however, this was determined in the registry study. We used the 'subsequent years' values to ensure a conservative analysis, as these were consistently lower. The analysis used a human capital approach.

Table H. Absences from work due to complications.

Complication	Mean number of days absent	Proportion of working year absent (% of 250 days)
Non-proliferative retinopathy	0	0
Proliferative retinopathy	0	0
Macular oedema	0	0
Severe visual impairment	0	0
Symptomatic neuropathy	17	7
Peripheral vascular disease	20	8
Amputation	36	14
Microalbuminuria	0	0
Macroalbuminuria (clinical nephropathy)	0	0
Nephropathy (uraemia stage)	21	8
Ischaemic heart disease	15	6
Myocardial infarction	19	8
Stroke	34	14
Heart failure	6	2

Data for the mean number of days absent were derived from a registry-based analysis of the impact of complications on absenteeism from work [26]. Means are expressed for the 14,746 patients in the analysis who were working. Absenteeism has been set to zero for complications not included in this article.

Health utilities
Various factors impacted on QALYs as described below.

Patient demographics

The model takes into account patient ages, genders, diabetes diagnoses and durations of diabetes for calculating utility. Utility scores used in the analyses are shown in **Table I**. However, as the values are the same for the two treatment arms in the analyses, they did not affect incremental analyses.

Table I. Demographic utilities.

Characteristic	Utility score	Reference
Age (per 10 years)	–0.024	[27]
Sex (women)	–0.056	[28]
Diabetes diagnosis	0.817	[28]
Duration of diabetes (per 10 years)	–0.0010	[28]

<u>Treatment</u>

Studies have shown that complex treatment regimens have a negative effect on treatment adherence [29–31]. Prandial (bolus) insulin in combination with a basal insulin is considered a complex regimen [32] as it requires multiple daily injections, different pen devices, frequent blood-glucose tests and complex titration schedules. The Global Attitudes of Patients and Physicians in Insulin Therapy study showed that the number of injections and the need to administer insulin at specific times or with meals were commonly reported issues with insulin therapy [29].

Table J shows the utility scores associated with the complexities of the different treatment regimens that were used in the present cost-effectiveness analyses. Data were derived from two time trade-off studies [33,34].

Table J. Impact of treatment complexity on utility scores.

	**Utility score**	**Reference**
Flexibility in the time for treatment (IDegLira)	0.015	[33]
One versus two injections daily (IDegLira versus GLP-1RA added to basal insulin)	0.015	[34]
One versus four injections daily, including planning* (IDegLira versus basal–bolus insulin regimen)	0.109	[34]
Self-monitoring of blood glucose†		
Once daily	–0.008	[33]
Four-times daily	–0.031	[33]

A study involving individuals from Sweden, Canada and the UK provided utility scores for 'flexibility in the time of treatment' (all patients had diabetes) and 'self-monitoring of blood glucose' (individuals with or without diabetes [33]). The remaining utility scores are from the Swedish cohort (all patients with T2D) in a multinational study [34]. *Patients are required to plan doses and food intake to maintain blood-glucose values at an appropriate level. †Calculated from the reported disutility of 0.0000221 per test.

Clinical factors

The utility scores associated with various clinical factors that were used in the cost-effectiveness analyses are shown in **Table K**. The utility score used for HbA1c levels was similar to that reported for type 1 diabetes [35]. Various studies have investigated how patient utility is affected by body mass index [27,34,36]. The utility score used in the analyses presented here was the lowest of those reported, with an alternative value (–0.021) [34] used in sensitivity analyses. Utility scores for daytime, rather than nocturnal, hypoglycaemia were used partly because this results in a conservative estimate and partly because such episodes are more common [37]. Data were derived from an extensive web-based time trade-off study carried out in Sweden, Canada, Germany, the UK and the USA [38]. QALY weights were then multiplied by the number of hypoglycaemia episodes in each patient and treatment arm.

Table K. Impact of clinical factors on patient utility in Swedish patients with T2D.

	Utility score	Reference
Glycated haemoglobin, %	–0.025	[34]
Body mass index, kg/m^2	–0.006	[27, 28]
Mild daytime hypoglycaemia*	–0.00449	[38]
Severe daytime hypoglycaemia*	–0.05250	[38]

Data are annual change in utility score per unit increase or event. *Values are for the cohort of Swedish patients with T2D (data on file) in the five-country analysis reported by Evans *et al*., 2013 [38]. T2D, type 2 diabetes.

Complications

Over time, many patients with diabetes develop complications that affect their quality of life. The utility scores associated with various diabetes-related complications that were used in the cost-effectiveness analyses are shown in **Table L**.

Table L. Impact of complications on patient utility.

Characteristic	Utility score	Reference
Retinopathy		
Non-proliferative retinopathy	–0.012	[28]
Proliferative retinopathy	–0.012	[28]
Macular oedema	–0.012	[28]
Severe visual impairment	–0.057	[27]
Neuropathy		
Symptomatic neuropathy	–0.084	[27]
Peripheral vascular disease	–0.061	[27]
Amputation	–0.272	[27]
Nephropathy		
Microalbuminuria	0.000	[27]
Clinical nephropathy (proteinuria)	–0.048	[27]
ESRD (uraemia stage)	–0.175	[27]
Macrovascular complications		
Ischaemic heart disease	–0.052	[28]
Myocardial infarction	–0.022	[28]
Stroke	–0.111	[28]
Heart failure	–0.082	[28]

ESRD, end-stage renal disease

Example of a utility calculation for year 1

Most variables in the utility calculation for year 1 were the same between liraglutide added to basal insulin and lixisenatide added to basal insulin: demographics, number of injections, number of blood sugar tests, flexibility, hypoglycaemia. Disutility due to complications occurs later.

Two variables differ between treatments: short-term utility due to HbA1c decrease (0.0025 per %-unit) and utility due to weight loss (0.006 per unit BMI).

The utility gain for year 1 due to HbA1c decrease and weight loss in the two treatment arms was calculated as follows: (HbA1c decrease x 0.025) + (BMI decrease x 0.006). Thus:
Liraglutide + basal insulin: (1.32 x 0.025) + (1.29 x 0.006) = 0.0401
Lixisenatide + basal insulin: (0.43 x 0.025) + (0.65 x 0.006) = 0.0147

Table M. Standard error sources and assumptions for the PSA.

**Variable**	**Source or assumption for SE**
Micro- and macrovascular complications: Costs	Assumption: SE=10%
Micro- and macrovascular complications: QALYs	Neuropathy and nephropathy: Bagust & Beale, 2004 [27]<br>Others: Assumption: SE=10%
Hypoglycaemia: Costs	Assumption: SE=20%
Hypoglycaemia: QALYs	Values taken from Evans *et al.*, 2013 [33]
Drug prices	Assumption: SE=0%
Treatment effects (HbA1c, BMI, etc) Liraglutide vs. lixisenatide	Values from Ahmann *et al.*, 2015 [7] and Riddle *et al.*, 2013 [39]
Treatment-related patient utility	Assumption: SE=50%

BMI, body mass index; HbA1c, glycated haemoglobin; PSA, probabilistic sensitivity analysis; QALY, quality-adjusted life year; SE, standard error

References

1. Ahmad Kiadaliri A, Gerdtham UG, Nilsson P, Eliasson B, Gudbjornsdottir S, Carlsson KS. Towards renewed health economic simulation of type 2 diabetes: risk equations for first and second cardiovascular events from Swedish register data. PLoS One. 2013; 8(5):e62650. doi: 10.1371/journal.pone.0062650. PMID: 23671618; PMCID: PMC3650043.
2. Bagust A, Hopkinson PK, Maier W, Currie CJ. An economic model of the long-term health care burden of Type II diabetes. Diabetologia. 2001; 44(12):2140-55. doi: 10.1007/s001250100023. PMID: 11793015.
3. Brown JB, Russell A, Chan W, Pedula K, Aickin M. The global diabetes model: user friendly version 3.0. Diabetes Res Clin Pract. 2000; 50 Suppl 3:S15-46. PMID: 11080561.
4. Clarke PM, Gray AM, Briggs A, Farmer AJ, Fenn P, Stevens RJ, et al. A model to estimate the lifetime health outcomes of patients with type 2 diabetes: the United Kingdom Prospective Diabetes Study (UKPDS) Outcomes Model (UKPDS no. 68). Diabetologia. 2004; 47(10):1747-59. doi: 10.1007/s00125-004-1527-z. PMID: 15517152.
5. Eastman RC, Javitt JC, Herman WH, Dasbach EJ, Zbrozek AS, Dong F, et al. Model of complications of NIDDM. I. Model construction and assumptions. Diabetes Care. 1997; 20(5):725-34. PMID: 9135934.
6. Hayes AJ, Leal J, Gray AM, Holman RR, Clarke PM. UKPDS outcomes model 2: a new version of a model to simulate lifetime health outcomes of patients with type 2 diabetes mellitus using data from the 30 year United Kingdom Prospective Diabetes Study: UKPDS 82. Diabetologia. 2013; 56(9):1925-33. doi: 10.1007/s00125-013-2940-y. PMID: 23793713.
7. Ahmann A, Rodbard HW, Rosenstock J, Lahtela JT, de Loredo L, Tornoe K, et al. Efficacy and safety of liraglutide versus placebo added to basal insulin analogues (with or without metformin) in patients with type 2 diabetes: a randomized, placebo-controlled trial. Diabetes Obes Metab. 2015; 17(11):1056-64. doi: 10.1111/dom.12539. PMID: 26179619.
8. Riddle MC, Aronson R, Home P, Marre M, Niemoeller E, Miossec P, Ping L, Ye J, Rosenstock J. Adding once-daily lixisenatide for type 2 diabetes inadequately controlled by established basal insulin: a 24-week, randomized, placebo-controlled comparison (GetGoal-L). Diabetes Care. 2013; 36(9):2489-96. doi: 10.2337/dc12-2454. PMID: 23628617.
9. NICE Decision Support Unit. Evidence Synthesis TSD series, TSD 2: A general linear modelling framework for pair-wise and network meta-analysis of randomised controlled trials (last updated Sept 2016); WinBUGS system(.odc) files (last updated Sept 2016). 2016. Available from: http://scharr.dept.shef.ac.uk/nicedsu/technical-support-documents/evidence-synthesis-tsd-series/. Accessed October 2017.
10. Gough SC, Bode B, Woo V, Rodbard HW, Linjawi S, Poulsen P, et al. Efficacy and safety of a fixed-ratio combination of insulin degludec and liraglutide (IDegLira) compared with its components given alone: results of a phase 3, open-label, randomised, 26-week, treat-to-target trial in insulin-naive patients with type 2 diabetes. Lancet Diabetes Endocrinol. 2014; 2(11):885-93. doi: 10.1016/S2213-8587(14)70174-3. PMID: 25190523.
11. Freemantle N, Mamdani M, Vilsboll T, Kongso JH, Kvist K, Bain SC. IDegLira versus alternative intensification strategies in patients with type 2 diabetes inadequately controlled on basal insulin therapy. Diabetes Ther. 2015; 6(4):573-91. doi: 10.1007/s13300-015-0142-y. PMID: 26582052.
12. Tandvårds- och läkemedelsförmånsverket (TLV) [Dental and pharmaceutical benefits board]. Databas för beslut [Database for decisions]. Stockholm, Sweden: Tandvårds- och läkemedelsförmånsverket (TLV) [Dental and pharmaceutical benefits board] 2013. Available from: https://www.tlv.se/beslut/sok/lakemedel/. Accessed July 2016.

13. World Health Organization Collaborating Centre for Drug Statistics Methodology. Definition and general considerations 2016. Available from: http://www.whocc.no/ddd/definition_and_general_considera/.
14. Jonsson L, Bolinder B, Lundkvist J. Cost of hypoglycemia in patients with Type 2 diabetes in Sweden. Value Health. 2006; 9(3):193-8. doi: 10.1111/j.1524-4733.2006.00100.x. PMID: 16689714.
15. Brod M, Christensen T, Thomsen TL, Bushnell DM. The impact of non-severe hypoglycemic events on work productivity and diabetes management. Value Health. 2011; 14(5):665-71. doi: 10.1016/j.jval.2011.02.001. PMID: 21839404.
16. Geelhoed-Duijvestijn PH, Pedersen-Bjergaard U, Weitgasser R, Lahtela J, Jensen MM, Ostenson CG. Effects of patient-reported non-severe hypoglycemia on healthcare resource use, work-time loss, and wellbeing in insulin-treated patients with diabetes in seven European countries. J Med Econ. 2013; 16(12):1453-61. doi: 10.3111/13696998.2013.852098. PMID: 24144009.
17. Södra regionvårdsnämnden. Regionala priser och ersättningar för södra regionvårdsnämnden. 2014. Available from: https://www.skane.se/Upload/Webbplatser/Sodra%20regionvardsnamnden/prislista/2014/helaprislistan2014.pdf.
18. Statistics Sweden. Salary structures, whole economy, Average monthly salary by sector 1992–2015: Statistics Sweden. Available from: http://www.scb.se/en/finding-statistics/statistics-by-subject-area/labour-market/wages-salaries-and-labour-costs/salary-structures-whole-economy/pong/tables-and-graphs/average-monthly-salary-by-sector-19922015/.
19. Steen Carlsson K, Persson U. Cost-effectiveness of add-on treatments to metformin in a Swedish setting: liraglutide vs sulphonylurea or sitagplitin. J Med Econ. 2014; 17(9):658-69. doi: 10.3111/13696998.2014.933110. PMID: 24950434.
20. Persson U, Willis M, Odegaard K. A case study of ex ante, value-based price and reimbursement decision-making: TLV and rimonabant in Sweden. Eur J Health Econ. 2010; 11(2):195-203. doi: 10.1007/s10198-009-0166-1. PMID: 19639352.
21. Apoteket. Produkter (Products) 2013. Available from: https://www.apoteket.se/produkt/gabapentin-teva-filmdragerad-tablett-800-mg-100-tabletter-blister-219568/.
22. Prompers L, Huijberts M, Schaper N, Apelqvist J, Bakker K, Edmonds M, et al. Resource utilisation and costs associated with the treatment of diabetic foot ulcers. Prospective data from the Eurodiale Study. Diabetologia. 2008; 51(10):1826-34. doi: 10.1007/s00125-008-1089-6. PMID: 18648766.
23. Sveriges kommuner och landsting. Sveriges kommuner och landsting, KPP-databasen, 2011. 2011. Available from: http://www.skl.se/vi_arbetar_med/statistik/kostnad-per-patient.
24. Gerdtham UG, Clarke P, Hayes A, Gudbjornsdottir S. Estimating the cost of diabetes mellitus-related events from inpatient admissions in Sweden using administrative hospitalization data. Pharmacoeconomics. 2009; 27(1):81-90. doi: 10.2165/00019053-200927010-00008. PMID: 19178126.
25. Ghatnekar O, Willis M, Persson U. Cost-effectiveness of treating deep diabetic foot ulcers with Promogran in four European countries. J Wound Care. 2002; 11(2):70-4. doi: 10.12968/jowc.2002.11.2.26675. PMID: 11901743.
26. Sørensen J, Ploug UJ. The Cost of Diabetes-Related Complications: Registry-Based Analysis of Days Absent from Work. Econ Res Int. 2013; 2013:1-8. doi: 10.1155/2013/618039.
27. Bagust A, Beale S. Modelling EuroQol health-related utility values for diabetic complications from CODE-2 data. Health Econ. 2005; 14(3):217-30. doi: 10.1002/hec.910. PMID: 15386666.
28. Kiadaliri AA, Gerdtham UG, Eliasson B, Gudbjornsdottir S, Svensson AM, Carlsson KS. Health utilities of type 2 diabetes-related complications: a cross-sectional study in Sweden. Int J Environ Res

Public Health. 2014; 11(5):4939-52. doi: 10.3390/ijerph110504939. PMID: 24810579; PMCID: PMC4053909.
29. Peyrot M, Barnett AH, Meneghini LF, Schumm-Draeger PM. Insulin adherence behaviours and barriers in the multinational Global Attitudes of Patients and Physicians in Insulin Therapy study. Diabet Med. 2012; 29(5):682-9. doi: 10.1111/j.1464-5491.2012.03605.x. PMID: 22313123; PMCID: PMCPMC3433794.
30. Rubin RR. Adherence to pharmacologic therapy in patients with type 2 diabetes mellitus. Am J Med. 2005; 118 Suppl 5A:27S-34S. doi: 10.1016/j.amjmed.2005.04.012. PMID: 15850551.
31. Saini SD, Schoenfeld P, Kaulback K, Dubinsky MC. Effect of medication dosing frequency on adherence in chronic diseases. Am J Manag Care. 2009; 15(6):e22-33. PMID: 19514806.
32. Inzucchi SE, Bergenstal RM, Buse JB, Diamant M, Ferrannini E, Nauck M, et al. Management of hyperglycemia in type 2 diabetes, 2015: a patient-centered approach: update to a position statement of the American Diabetes Association and the European Association for the Study of Diabetes. Diabetes Care. 2015; 38(1):140-9. doi: 10.2337/dc14-2441. PMID: 25538310.
33. Evans M, Jensen HH, Bogelund M, Gundgaard J, Chubb B, Khunti K. Flexible insulin dosing improves health-related quality-of-life (HRQoL): a time trade-off survey. J Med Econ. 2013; 16(11):1357-65. doi: 10.3111/13696998.2013.846262. PMID: 24111563.
34. Ridderstrale M, Evans LM, Jensen HH, Bogelund M, Jensen MM, Ericsson A, et al. Estimating the impact of changes in HbA1c, body weight and insulin injection regimen on health related quality-of-life: a time trade off study. Health Qual Life Outcomes. 2016; 14:13. doi: 10.1186/s12955-016-0411-0. PMID: 26801908; PMCID: PMCPMC4722746.
35. McQueen RB, Ellis SL, Maahs DM, Anderson HD, Nair KV, Libby AM, et al. Association between glycated hemoglobin and health utility for Type 1 diabetes. Patient. 2014; 7(2):197-205. doi: 10.1007/s40271-014-0045-4. PMID: 24458545.
36. Lane S, Levy AR, Mukherjee J, Sambrook J, Tildesley H. The impact on utilities of differences in body weight among Canadian patients with type 2 diabetes. Curr Med Res Opin. 2014; 30(7):1267-73. doi: 10.1185/03007995.2014.899207. PMID: 24588550.
37. Ostenson CG, Geelhoed-Duijvestijn P, Lahtela J, Weitgasser R, Markert JM, Pedersen-Bjergaard U. Self-reported non-severe hypoglycaemic events in Europe. Diabet Med. 2014; 31(1):92-101. doi: 10.1111/dme.12261.
38. Evans M, Khunti K, Mamdani M, Galbo-Jorgensen CB, Gundgaard J, Bogelund M, et al. Health-related quality of life associated with daytime and nocturnal hypoglycaemic events: a time trade-off survey in five countries. Health Qual Life Outcomes. 2013; 11:90. doi: 10.1186/1477-7525-11-90. PMID: 23731777; PMCID: PMCPMC3679729.
39. Riddle MC, Aronson R, Home P, Marre M, Niemoeller E, Miossec P, et al. Adding once-daily lixisenatide for type 2 diabetes inadequately controlled by established basal insulin: a 24-week, randomized, placebo-controlled comparison (GetGoal-L). Diabetes Care. 2013; 36(9):2489-96. doi: 10.2337/dc12-2454. PMID: 23628617; PMCID: PMC3747925.
